# Photocatalytic Seawater Splitting by Earth‐Abundant Catalysts: Metal‐Semiconductor Metamaterials Made of Plasmonic Magnesium Diboride and Transitional Metal Dichalcogenides

**DOI:** 10.1002/chem.202403050

**Published:** 2024-11-14

**Authors:** Hongkai Zhou, Alexander N. Grigorenko, Vasyl G. Kravets

**Affiliations:** ^1^ Department of Physics and Astronomy the University of Manchester Manchester M13 9PL UK

**Keywords:** Seawater splitting, Plasmonic photocatalyst, Green hydrogen, Magnesium diboride, Transitional metal dichalcogenides

## Abstract

Metal‐semiconductor metamaterials hold great promise for photocatalytic water splitting due to their excellent light harvesting in a broad spectral range as well as efficient charge carrier generation and transfer. In the majority of such metamaterials, semiconductors are used to initiate the water splitting reaction, while their metal counterparts are employed to improve light harvesting through plasmonic effects. Here, we describe for the first time an exceptional reversed case of metal‐semiconductor photocatalysts in which metals are used to initiate the water splitting reaction and semiconductors are employed to improve light harvesting through the blackbody effect and serve as co‐catalysts. The studied photoanodes are made of non‐noble plasmonic MgB_2_ combined with transition metal dichalcogenides (TMDCs). The plasmonic resonances of the MgB_2_ component contribute to field confinement, plasmon–exciton coupling, and hot‐electron transfer providing an enhancement of photoactivity in the entire solar spectrum capable of water splitting. The TMDC component provides impedance matching and enhances light absorption by the metal catalyst. We demonstrate seawater splitting with MgB_2_‐TMDCs photoanodes attaining current densities of ~3 mA cm^−2^ at solar radiation. The overall efficiency of hydrogen production in seawater splitting by sunlight with the help of the studied photoanodes is 3 % at a bias voltage of *V_bias_* =0.3 V.

## Graphical abstract

Novel photocatalysts for efficient seawater splitting made from metal‐semiconductor hybrid metamaterials are studied in this work. The Earth‐abundant metal MgB_2_ is employed as main photocatalyst and the TMDs as the co‐catalyst, among which MgB_2_/WS_2_ shows the largest enhancement of seawater splitting compared to the separate elements. An efficient mechanical fabrication method is employed to produce MgB2‐TMDCs hybrid photoanodes.

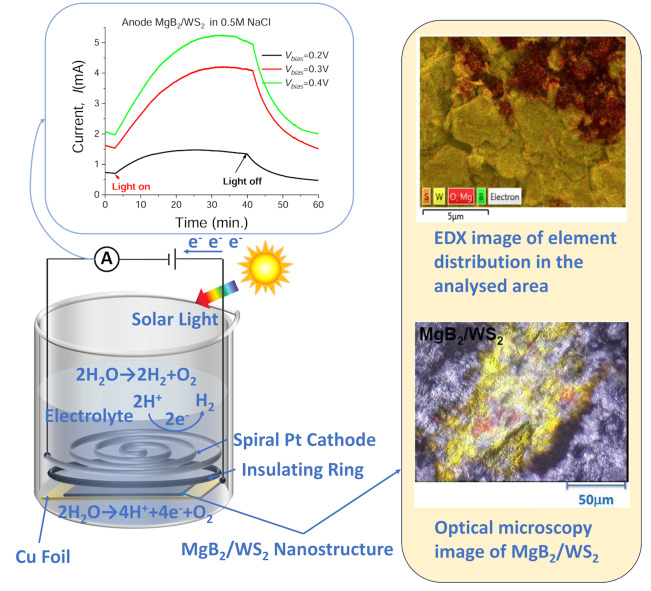

## Introduction

Recently, a whole variety of photocatalytic systems based on new physical mechanisms has been suggested. Among them, plasmonic photocatalysts show a great promise due to their high solar energy utilization efficiency. Indeed, plasmonic nanostructures can efficiently absorb solar light through localized surface plasmon resonances (LSPRs) and then convert it into hot electrons that drive redox reactions.[^1^, ^2^, ^3^, ^4^, ^5^, ^6^, ^7^, ^8^, ^9^, ^10^, ^11^] The process of energy transfer from plasmonic nanostructures to reactants plays a decisive role in the overall photocatalytic performance. However, the underlying physical mechanisms of plasmonic photocatalytic activity are still not clear despite being of great scientific and technological interests. This has led to plasmonic catalysts becoming one of the hottest topics of modern research.[^3^, ^6^, ^9^]

Linic's group in 2011 was first to observe direct molecular oxygen activation and ethylene epoxidation on plasmonic Ag nanoparticles^[1]^ which triggered an interest in the plasmonic photocatalysts.[^12^, ^13^] Halas et al. demonstrated that H_2_ could be dissociated on plasmonic Au nanoparticles under light excitation without the need for external heating due to the involvement of hot electrons in the catalysis.^[2]^ It was also shown that plasmon‐induced photo‐chemistry can be enhanced by multilayer interference effects which promotes high concentration of sunlight close to the electrode/liquid interface.^[11]^ Despite ever increasing interest in developing pure plasmonic photo‐catalysts, their efficiency is still very low (∼0.1 %).^[14]^

In the early stages, more efficient plasmonic photocatalysts for water splitting were fabricated by combining plasmonic metal nanostructures with wide bandgap semiconductors (for example, TiO_2_), where semiconductors were used to drive the water splitting reaction while noble metal nanoparticles (NPs) were used to improve light harvesting through plasmonic effects.[^12^, ^13^] It was shown that a dense array of aligned gold nanorods capped with TiO_2_ could produce 20 times higher efficiency as compared to that of pure TiO_2_ films.^[4]^ Later, transitional metal dichalcogenides (TMDCs) have received a lot of attention as promising photocatalysts for water splitting due to the fact that the bandgap energy of TMDCs can be varied by controlling the number of stacked TMDC layers or TMDC composition.^[15]^ In addition, TMDCs demonstrate high optical absorption coefficients up to one order of magnitude greater than conventional direct bandgap semiconductors promoting efficient solar energy conversion to hydrogen. In fact, ultrathin (<20 nm) TMDCs can achieve near‐unity, broadband, and omnidirectional absorption in the visible light spectrum.^[16]^ The wide range of TMDC band gaps (~1.0–2.0 eV) are also well suited for highly efficient water splitting.^[17]^ Recently, MoS_2_ has been reported to be a good catalyst for both photocatalytic and electro‐catalytic H_2_ evolution reactions due to the existence of abundant exposed edges, and the active sites at the S edges of MoS_2_ crystal layers.^[18]^ Moreover, plasmonic‐metal/2D‐semiconductor hybrids showed a significant promise for constructing high‐performance photo‐catalysis devices due to the fact that the hybrid systems can solve the problems related to light absorption, charge separation and migration, and surface redox reactions.[^19^, ^20^] It was shown that a combination of different 2D materials (e. g., anatase TiO_2_ nano‐sheets and layered MoS_2_) can strongly enhance the photocatalytic activity of such hybrid photo‐catalyst due to the increased contact interface and charge transfer rate.^[21]^ L. Guo et al.^[22]^ suggested to use non‐metal plasmonic MoS_2_/TiO_2_ heterostructures for efficient photocatalytic H_2_ generation. In these heterostructures, plasmon resonances associated with MoS_2_ coatings effectively provide charge transfer to the TiO_2_ matrix which is important for the solar energy harvesting and photocatalytic H_2_ production.

In our previous works, we have shown that MgB_2_ can be an efficient all‐metal photo‐catalyst for water splitting[^23^, ^24^] due to its specific surface properties and the presence of localised plasmons. We also demonstrated water splitting by other metal diboride (in addition to Earth‐abundant MgB_2_) by verifying photo‐catalytic solar light conversion to hydrogen. Unfortunately, metals have a large impedance mismatch with air and water which leads to large light reflection from metallic photoanodes. As a result, overall efficiency of light to hydrogen conversion in our previous works was not large. In this work, to address the issue of the impedance mismatch, we have used ideas of plasmonic blackbodies described in.[^25^, ^26^]

Here we report highly efficient room‐temperature seawater splitting by composite photoanodes made of metal‐dielectric metamaterial comprised of magnesium diboride (MgB_2_) nano‐sheets combined with TMDC nano‐flakes (MgB_2_‐TMDCs) under the light of a solar simulator. In contrast to previously studied metal‐semiconductor structures, our metamaterials make use of metals (MgB_2_) in order to initiate the water splitting reaction while semiconductor counterparts (TMDCs) are employed to improve light harvesting (through the blackbody effect) and to serve as a co‐catalyst. Strong stacking between MgB_2_ and TMDCs is conditioned by the layered structure of the components and van der Waals interactions. We propose a new simple method of fabricating hybrid MgB_2_‐TMDCs nanostructures using a mechanical rolling mill procedure. We show that inexpensive MgB_2_/MoS_2_, MgB_2_/MoSe_2_, and MgB_2_/WS_2_ photoanodes provide efficient plasmonic seawater splitting and could be a viable alternative to pure semiconductor catalysts. The suggested architecture of a photoanode is important for fast interfacial charge separation and broadband absorption of solar light.

The usage of seawater for effective production of hydrogen is one of the most important results of our work. Indeed, water becomes an increasingly crucial resource in the world. Due to population growth, the availability of fresh water as a feedstock becomes problematic. Hence, the usage of abundant seawater for industrial production of H_2_ is beneficial as the oceans and seas represent 96.5 % of the total water reserves of the Earth providing an almost unlimited resource.[^27^, ^28^] Nowadays, the best strategy to produce H_2_ from seawater is to split it into hydrogen and oxygen using electricity that comes from renewable energy sources, such as photovoltaic cells. Our work allows direct conversion of sunlight into hydrogen using seawater splitting by MgB_2_‐TMDCs hybrid nanostructures. We also confirm that metal borides combining with TMDCs layered flakes are efficient and stable for seawater splitting which was recently predicted for boron based nanocomposites in.^[29]^

## Experiments and Results

### Sample Fabrication

The layered structure of MgB_2_, MoS_2_, MoSe_2_ and WS_2_ materials as well as MgB_2_‐TMDC composite materials implies that they can be exfoliated into thin nanosheets assembly down to a monolayer.[^30^, ^31^] In order to fabricate samples for our study, we utilized the Pepetools 90 mm Flat Rolling Mill. There exist many useful methods to produce composite hybrid structures. These include solvothermal, ionothermal, microwave‐assisted, sonochemical, mechanochemical, photochemical, radiation‐induced, plasma‐induced, and organic flux synthesis^32^. In comparison to a solvothermal synthesis (which is also based on elevated pressure as well as on elevated temperature and proper solvents), the suggested mechanical rolling mill procedure is simple, cheap and reliable. It does not require high temperatures and solvents. It can be used in mass‐production of stable photoanodes on flexible large‐size substrates providing photo‐anodes with multiwavelength localised surface plasmon resonances (LSPRs). In the rolling mill procedure, we used MgB_2_ and TMDC powders from Sigma Aldrich with purity of over 99 %. Cu foils with thickness of <100 μm were used as the substrates. The powders of MgB_2_ and TMDCs were additionally grinded. The mechanical rolling mill procedure was performed as follows: (i) a 20 mm×20 mm square surface of the Cu foil was covered with chosen MgB_2_‐TMDC mixed powder with weight of ~30 mg and then the top of the stack was covered by a second Cu foil of the same size. (ii) The resulting stack was passed multiple times (about 10) through a rolling mill, with increasing pressure applied between rolls in order to obtain thin hybrid MgB_2_‐TMDC films. (iii) Finally, the longitudinal expansion of the samples by a factor of ~2 times was achieved. By leveraging the capabilities of the rolling mill and utilizing high‐quality powders, we were able to fabricate samples with nice structural and optical properties (strong absorption in the visible–near IR ranges). We found that rolling mill conditions could affect the final shape and size of MgB_2_‐TMDC nanostructures. The short milling time was not enough to cause sufficient adhesion to the substrate, while a longer milling time increased the nanostructure size of TMDC part due to ductility of TMDC structures. Therefore, the choice of milling conditions is important to control the physical and chemical properties of MgB_2_‐TMDC nanostructures. MgB_2_‐TMDs hybrid nanostructures were prepared by mixing MgB_2_ powder with 33, 50 and 67 wt. % of MoS_2_ (MoSe_2_,WS_2_) flakes. The maximal photocatalytic efficiency was be achieved for compounds of approximately equal masses of MgB_2_ and TMDC materials. Due to this fact, we focuss below on the properties of 50 % wt. MgB_2_‐TMDC metamaterials.

**Figure 1:**
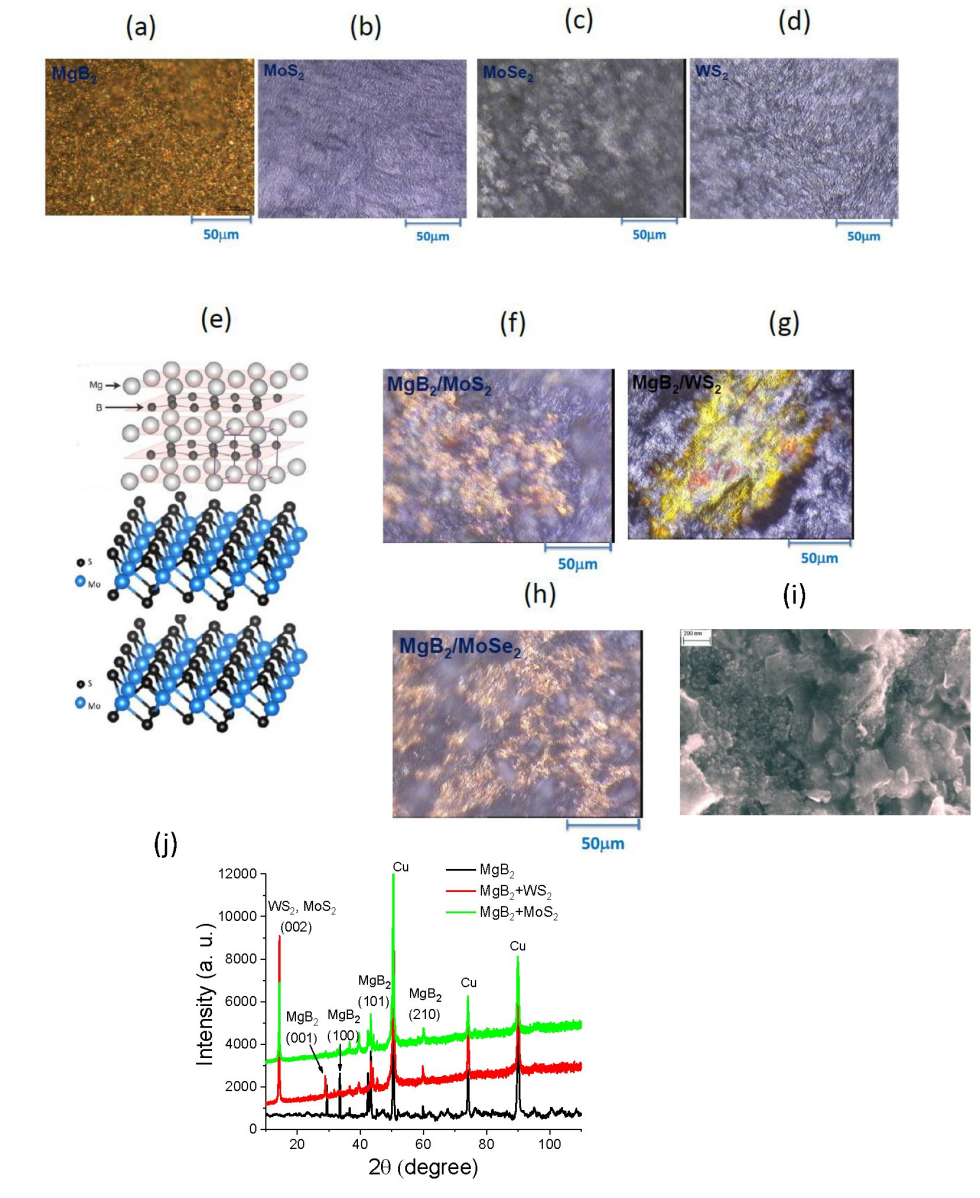
Optical microscopy and scanning electron microscopy (SEM) of stable MgB_2_‐TMDCs hybrid photocatalysts produced by a mechanical rolling mill procedure measured in a reflection phase‐contrast microscopy mode (crossed polarizers with an additional phase shift) (a‐d) Optical images of pure plasmonic MgB_2_ and 2D‐like semiconductors: MoS_2_, MoSe_2_ and WS_2_ layered nanostructures. (e) Schematic design of the integrated MgB_2_‐TMDCs nanostructured photo‐anodes. (f–h) Optical microscopy images showing the assembly of MgB_2_‐TMDCs metamaterials which exhibit different colours due to excitation of localised surface plasmon resonances. (i) A SEM image of MgB_2_‐WS_2_ composite. (j) XRD analysis of photo‐anodes.

Figures 1a–d show optical images of pressed indvidual flakes (MgB_2_, MoS_2_, MoSe_2_ and WS_2_) that were used for creation of hybrid devices. These images confirm the presence of mircometer‐size inhomogeneties/flakes. Figure 1e demonstrates the layered structures of MgB_2_ and TMDCs that provided van der Waals interactions between the components. Figures 1f–h show the optical images of fabricated hybrid MgB_2_‐TMDCs photo‐anodes. The presence of LSPRs in the fabricated MgB_2_‐TMDCs samples is confirmed by multiple colours changing from blue to red in the phase‐contrast optical images, see Figure 1f–h. These areas are randomly distributed on the sample surface. Localised surface plasmon resonances shown in Figure 1 indicate that the fabricated samples should be described as optical “metamaterials”. (Indeed, the presence of LSPRs implies that the sizes of MgB_2_ and TMDCs building blocks are comparable to interrogating light wavelength. This allowed us to modify light‐matter interaction and achieve stronger light absorption and more efficient photocatalytic water splitting than those achieved by individual elements.) The multitude of colours of fabricated MgB_2_‐TMDCs samples is due to the change of reflectivity induced by excitation of LSPRs. A high resolution scanning electron microscopy (SEM) image of MgB_2_‐WS_2_ composite is shown in Figure 1i. (SEM images for other composites MgB_2_‐MoS_2_ and MgB_2_‐MoSe_2_ look the same). The SEM image demonstrates that fabricated samples consist from randomly mixed 3D–like MgB_2_ blocks and flat 2D–like WS_2_ nanostructures. It is worth noting that MgB_2_‐WS_2_ nanostructures have a distribution of lateral dimensions from tens of nanometers up to micrometers (0.1–5 μm) and a distribution of thicknesses from a singular nanometer to tens of nanometers (1–30 nm). In addition, narrow gaps of few nanometers between different nanostructures were formed indicating a strong interaction between the plasmonic metals and TMDCs nanostructures. The thickness of fabricated MgB_2_‐TMDCs photo‐anodes was approximately in range of 100–130 nm measured with the help of the mechanical profilometer Dektakt 32.

The structural properties of the MgB_2_, MgB_2_/WS_2_ and MgB_2_/MoS_2_ photoanodes before and after seawater splitting experiments were determined by the X‐ray diffraction (XRD) technique using CuKα radiation (*λ*=0.15418 nm) in the range of angles 2*θ* from 10° to 110°. Figure 1(j) shows XRD spectra for the samples after seawater splitting experiments performed under V_bias_=0.3 V (the results before/after experiments were almost identical). Diffraction peaks were observed in the XRD pattern of the fabricated MgB_2_‐TMDCs samples associated with the MgB_2_ crystalline phases (Figure 1j): 2*θ*≈29.6° (001), 33.5° (100), 42.4° (101), 59.9° (110), which are in agreement with.^[33]^ Diffraction peaks at 2*θ≈*14.37° for MgB_2_/WS_2_ and MgB_2_/MoS_2_ photo‐anodes belong to the (002) planes of WS_2_ and MoS_2_ and are similar to the diffraction data for few TMDC exfoliated layers.^[34]^ Note that in the X‐ray diffraction spectra, only the (002) peaks usually are present at diffraction angles 2*θ*≈14.3° (MoS_2_), 2*θ*≈13.7° (MoSe_2_), and 2*θ*≈14.3° (WS_2_). This suggests that exfoliation did not affect the crystal structure or bonding configuration of MoS_2_, MoSe_2_ and WS_2_. The disappearance of other peaks in the XRD pattern of MgB_2_‐TMDCs photo‐anodes indicates that few‐layer TMDC nanosheets were successfully obtained through the milling fabrication process.^[34]^ Some additional peaks for MgB_2_‐TMDCs films on Cu substrate could correspond to the Cu crystalline phases.^[35]^

### Photo‐Catalytic Tests

**Figure 2:**
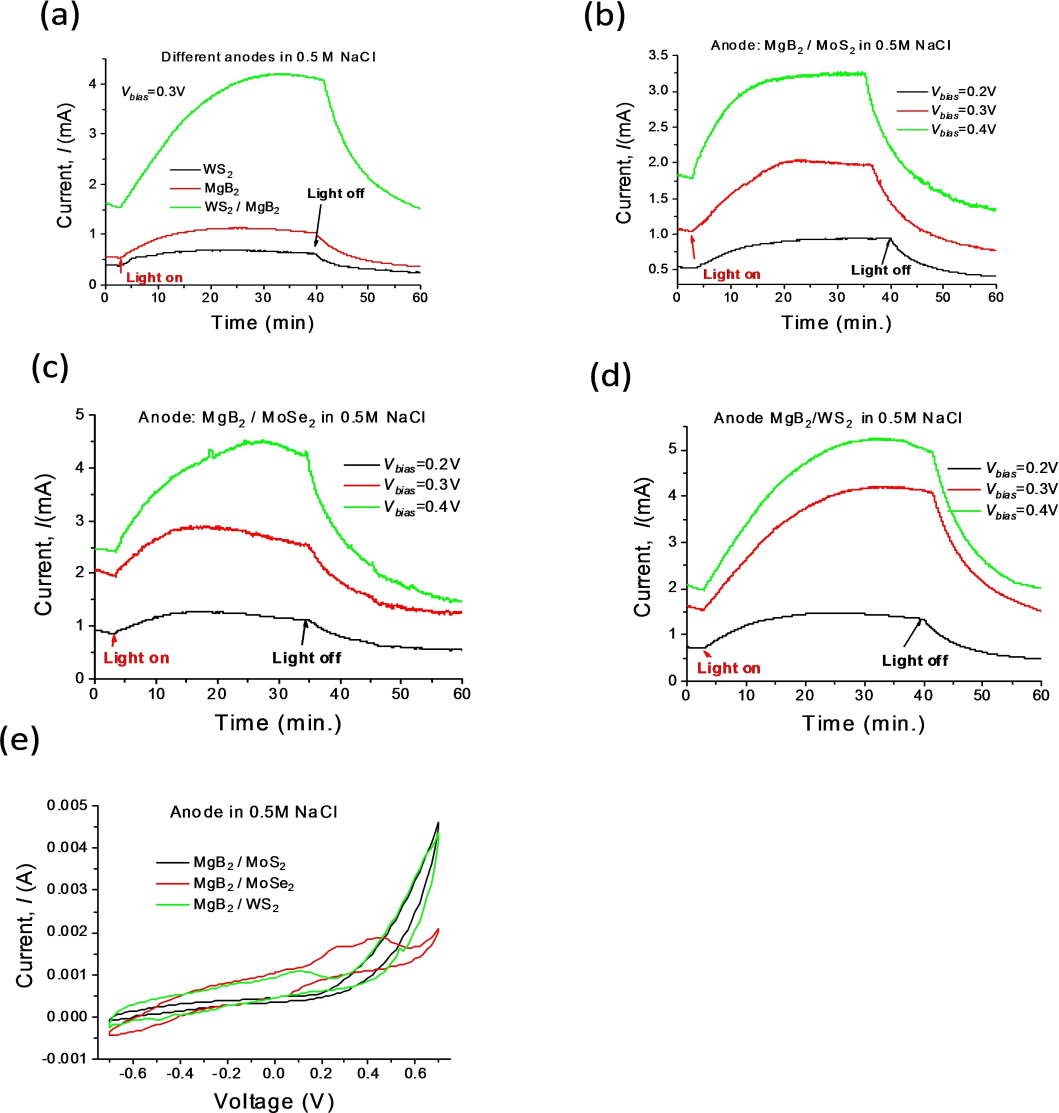
Seawater splitting: photocurrent as a function of time and external voltage, *V_bias_*, under illumination of a solar light simulator. (a) Photocurrents produced in PEC for pure WS_2_, MgB_2_ and MgB_2_/WS_2_ hybrid photoanodes in 0.5 M NaCl electrolyte under external voltage of *V_bias_* =0.3 V. (b) Photocurrents in a MgB_2_/MoS_2_ metamaterial in 0.5 M NaCl electrolyte as a function of *V_bias_*. (c) Photocurrents in MgB_2_/MoSe_2_ metamaterial in 0.5 M NaCl electrolyte as a function of *V_bias_*. (d) Photocurrents in MgB_2_/WS_2_ metamaterial in 0.5 M NaCl electrolyte as a function of *V_bias_*. (e) Cycling voltammetry (I−V characteristics) for MgB_2_‐TMDCs photoanodes.

We have tested the efficiency of MgB_2_‐TMDCs fabricated photoanodes in a two‐electrode photo‐electrochemical cell (PEC) configurations with a Pt wire serving as a cathode. Solar light was provided by a solar simulator AM 1.5 G (Newport) with illumination intensity of ∼100 mW cm^−2^. The current‐voltage (I−V) characteristics were recorded using a digital source meter (Keithley, Model 2400) with and without an external potential bias (*V_bias_*) applied across the cell. The one‐component MgB_2_ photo‐anodes generated photocurrents up to 1.2 mA under *V_bias_* =0.3 V in seawater (see Figure 2a) which we modelled as a solution of 0.5 M NaCl in distilled water (DI) (the measured pH of 0.5 M NaCl−DI water solution was around ∼7–8 in agreement with.^[36]^ It is convetional to use aqueous 0.5 M NaCl solution as a reasonable surrogate of natural seawater, see perspective.^[36]^) The bias voltage was measured with respect to reversible hydrogen electrode (RHE). The initial contact voltage between plasmonic MgB_2_‐TMDC photo‐anodes and Pt photo‐cathodes embedded in 0.5 M NaCl−DI electrolyte in the dark condition was found to be V_*init*_~0.7–0.9 eV. This contact voltage was responsible for relatively large dark currents in the photo‐catalytic experiments.

The sizes of the studied photoanodes were ∼1 cm^2^. The size of a Pt cathode was ~0.15 cm^2^ for all measurements with two electrodes separated by ∼3 mm. The changes of the photocurrent for MgB_2_/WS_2_ hybrid photo‐anodes induced by solar light were around ~2.7 mA/cm^2^. At the same time, the changes of the photocurrent induced by solar light were ~0.6 mA/cm^2^ for MgB_2_ photoanodes and ~0.3 mA/cm^2^ for WS_2_ photo‐anodes, see Figure 2a. Thus, photo‐induced changes of the current (which is the difference between maximal currents in illumination and dark conditions) for MgB_2_/WS_2_ hybrid photo‐anodes were 9 times larger than that for pure WS_2_ photo‐anodes and 4.5 times larger than that for pure MgB_2_ photo‐anodes. The changes in the cell current after switching on solar light were significantly enhanced for all the investigated MgB_2_‐TMDCs photo‐anodes, Figures 2a–d, as compared to those for pure TMDCs photoanodes (which were around ∼0.3–0.7 mA/cm^2^, see Figure 2a for pure WS_2_ photoanodes). We found that our inexpensive MgB_2_‐TMDCs photo‐anodes submerged into a 0.5 M NaCl−DI water solution under a small bias (0.2 V≤*V_bias_* ≤0.4 V) provide strong enhancement of photocatalytic water splitting. The water splitting reaction by MgB_2_‐TMDC was accompanied by bubbles formation on the surface of a photo‐anode (which generated O_2_) and a Pt cathode (which generated H_2_) which affected the PEC photocurrent leading to its fluctuations and a slight drop at a longer timescale. The bubbles were observable by the naked eyes (or in an optical microscope) under solar illumination. The overall volume of bubbles on the Pt wires was approximately two times larger than the overall volume of bubbles on the MgB_2_‐TMDC photo‐anode. In addition, the observations showed that the volume of produced gases corresponded to the measured photocurrent with 95 % of Faraday efficiency. As a result, the overall volume of bubbles on both electrodes strongly increased with an increase of the bias voltage (and hence the photocurrent). All these corroborate the presence of the water splitting reaction. It was found that MgB_2_‐WS_2_ photoanodes were stable and remained active after 60 hours of seawater splitting. In order to find the optimal value of the bias voltage, we used cycling voltammetry (CV), see Figure 2e, where we cycled the bias voltage applied to the investigated MgB_2_‐TMDCs photoanodes in the seawater (0.5 M NaCl electrolyte) linearly from the potential of −0.7 V to +0.7 V and in reverse with scan rate of 1 mV/s. We then chose the bias voltage corresponding to the points where the peak currents were observed (for example:∼0.27–0.3 V for MgB_2_/MoSe_2_). The current peaks on the CV plot indicate the transfer of electrons, and the voltage at which the peak occurs corresponds to a certain redox process.^[37]^

The light‐to‐photocurrent conversion can be described by incident photon to current conversion efficiency (IPCE) defined as:[^38^, ^39^]

(1) $$IPCE=\frac{J_{SC}\left(mA\;{cm}^{-2}\right)\times \left(V_{redox}-V_{bias}\right)}{P_{light}\left(mW\;{cm}^{-2}\right)}$$

where *J_SC_* is the short‐circuit difference in photocurrent density, *V_bias_* is the applied potential between photo‐electrode and counter electrode, *P*_*ligh*t_ is the total irradiation input, and the redox potential *V_redox_* is equal to 1.23 V for water splitting. The highest efficiency of ∼3 % (normalised to the photoanode area) for seawater splitting was achieved for MgB_2_/WS_2_ photoanodes under *V_bias_* =0.3 V. By studying the hydrogen gas produced, we confirmed 0.95 % Faraday efficiency of our cell.[^23^, ^24^] This implies that each 2 electrons generated ~0.95 molecules H_2_ and hence IPCE represents overall efficiency of light conversion to hydrogen with high accuracy.

### Ellipsometric Measurement of the Complex Refractive Index

The main incentive for combining metallic MgB_2_ and semiconductor TMDCs in hybrid photoanodes in our studies was to address the problem of impedance mismatch between the metal (MgB_2_) and dielectric (water) and, therefore, to improve light absorption by the hybrid photoanodes. To achieve this goal we made use of the theory of plasmonic blackbody.[^25^, ^26^] This theory suggests that for an artificial material of some thickness there exist optimum values of refractive index, *n*, and absorption coefficient, *k*, that generate the maximal light absorption in the material. These values are not easily attainable in pure materials; however, they could be achieved in artificial nanostructured or heterostructured compounds. To check whether we succeeded in realising plasmonic blackbody, we performed measurements of optical properties of the studied photoanodes and extracted their complex refractive indices.

**Figure 3:**
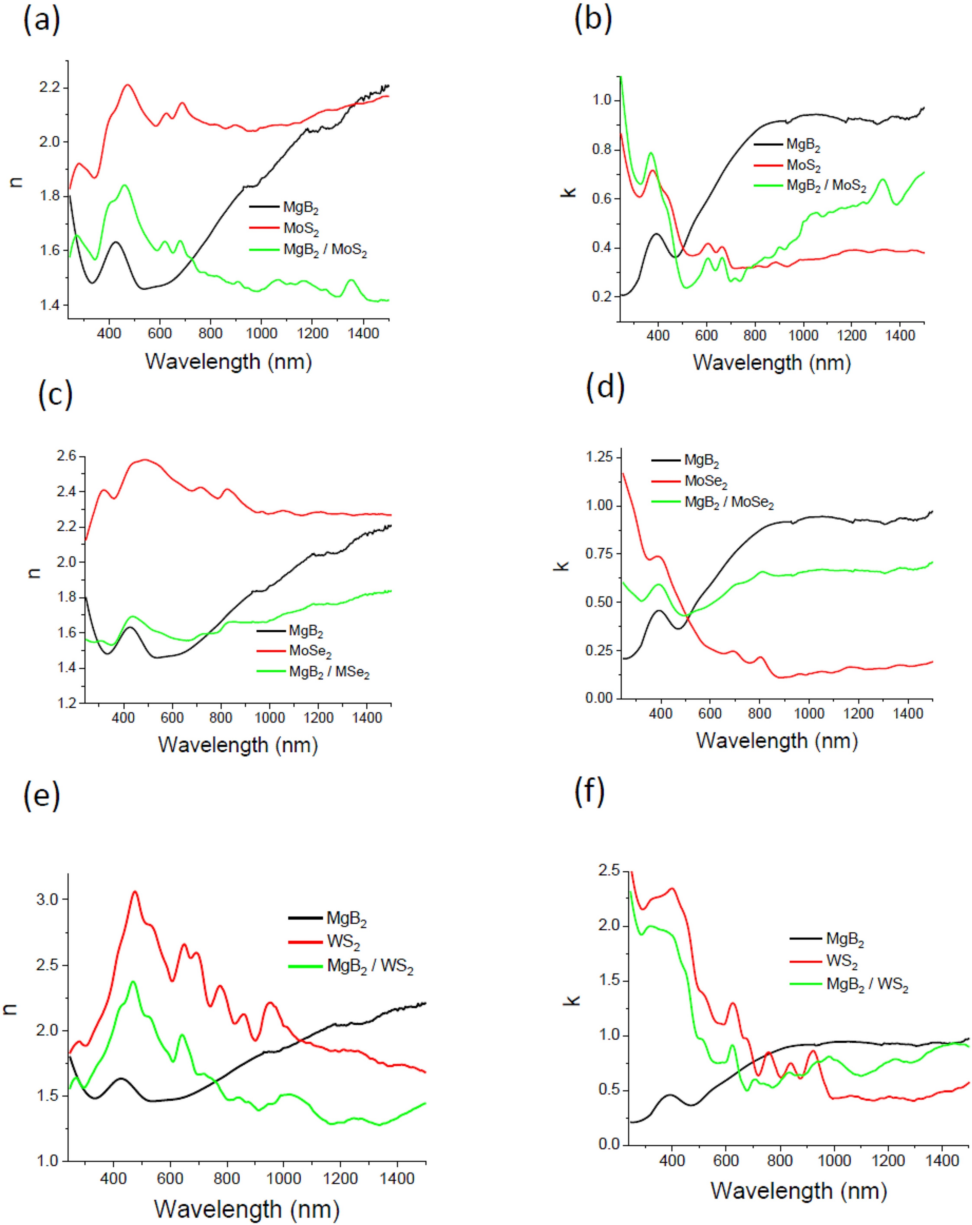
Optical properties of MgB_2_‐TMDCs nanostructured films. (a,b) The real and imaginary parts of the refractive index for pure MgB_2_, MoS_2_ and MgB_2_/MoS_2_ photoanodes. (c,d) The real and imaginary parts of the refractive index for pure MgB_2_, MoSe_2_ and MgB_2_/MoSe_2_ photoanodes. (e,f) The real and imaginary parts of the refractive index for pure MgB_2_, WS_2_ and MgB_2_/WS_2_ photoanodes.

We found that the hybrid MgB_2_‐TMDCs photoanodes indeed show superior absorption as compared to that of a single component photoanode made of MoS_2_, MoSe_2_ or WS_2_. To determine the optical absorption of fabricated MgB_2_‐TMDCs metamaterials, the ellipsometry was employed. We used a variable angle focussed‐beam spectroscopic ellipsometer Woollam M2000F in the wavelength range of 240–1500 nm. The spot size on the sample was approximately 30 μm×70 μm at ~70–80° angles of incidence. The ellipsometry essentially measures a ratio of polarized reflections. It yields two spectral parameters (*Ψ* and *Δ*) related to the amplitude (tan*Ψ*) and relative phase (*Δ*) of a complex reflectance ratio *ρ* that provides the ratio of the reflection coefficient for *p*‐polarised, *r_p_*, and *s*‐polarised, *r_s_*, light as *ρ=r*_p_/*r*_s_=(tan*Ψ*)exp(*iΔ*).[^40^, ^41^] The ellipsometric measurements were done at high angles of light incidence *θ_i_* ≈70–75°. These angles correspond to pseudo‐Brewster angles of the studied samples in visible light and provide the best accuracy in extraction of optical constants.^[40]^ To obtain values of the complex refractive index n*=*n*+*ik* for hybrid MgB_2_‐TMDCs photo‐anodes, we fit the ellipsometry data measured on the samples with Woollam M2000F ellipsometer using Wvase32 software where the studied photo‐anodes were modelled in effective medium approximation. The extracted real and imaginary parts of the refractive index n*=*n* +*ik* for all individual components and hybrid mixtures are shown in Figure 3. The absorption coefficient, *k*, of MgB_2_‐TMDCs composites show a combination of the spectral features of MgB_2_ and TMDCs, with TMDCs absorbing mainly in UV‐visible light while MgB_2_ absorbing in the near IR range. Compared to the pure TMDCs photo‐anodes, the absorption spectra of MgB_2_‐TMDCs hybrid photo‐anodes show an enhanced absorption in the visible – near IR spectral region ranging from 600 to 1500 nm – further increased with replacing Mo by W atoms (Figure 3), which is in accordance with the colour change of the samples from orange to yellow shown in Figure 1. Note that two spectral regions with dominant absorption variations (Figures 3b, d, f) are observed in the wavelength range of 550–900 nm where A, B excitons of different TMDCs show pronounced absorption and in the range of 350–500 nm that corresponds to C exciton.^[42]^ Thus, MgB_2_‐TMDCs hybrid materials can indeed enhance sunlight absorption resulting in higher photocatalytic activity.

The optical constants *n* and *k* of the studied photoanodes extracted with the help of spectroscopic ellipsometry show strong dispersion and absorption associated with A and B and C exciton in TMDCs and LSPR excitations in MgB_2_. Excitonic absorption peaks A and B which arise from direct gap transitions at the K point are found in agreement with the previous studies on the bulk material.^[43]^ We also found that MgB_2_‐TMDCs hybrid photo‐anodes display smaller values of the real part of the refractive index, *n*, in comparison to those of pure MoS_2_, MoSe_2_ or WS_2_ photo‐anodes. It is worth noting that *n* is ranged between 1.3 and 1.6 in the visible (*λ*>500 nm) and near IR region for MgB_2_‐TMDCs fabricated photo‐anodes, suggesting mostly dielectric response (Figure 3). In our previous works dedicated to plasmonic blackbody and perfect absorbers[^25^, ^26^] we showed that combination of *n*≈1 and small *k≈*0.5 provides almost total absorption of light in the air for a covering layer with thickness of *d<λ*. To realise blackbody behaviour[^25^, ^26^] (*aka* perfect absorber conditions) for a reasonably thick photoanode in water/seawater solutions, the real part of the refractive index should match condition *n≈N_wt_* ≈1.33 for the top layer (water) and the imaginary part, *k*, should be small to guarantee the absence of reflection from the first interface according to the Fresnel theory. Then, heaving a reasonably small imaginary part of the refractive index *k* would assure the total light absorption in the photo‐anode provided 2*πkd/λ*≫1, where *d* is thickness of a photo‐anode (hence the properties of the substrate would make no bearing on the light absorption). Such combination of *n* and *k* would provide blackbody‐like behaviour MgB_2_‐TMDCs hybrid photo‐anodes that will effectively absorb solar light in seawater. Figure 3 confirms that the effective optical constants extracted for MgB_2_‐TMDCs hybrid photo‐anodes in the wavelength range of maximal solar radiation (500–800 nm) are indeed close to the values of the effective constants that would guarantee the blackbody absorption in accordance with the Fresnel theory.[^25^, ^26^]

### FTIR Spectra

**Figure 4:**
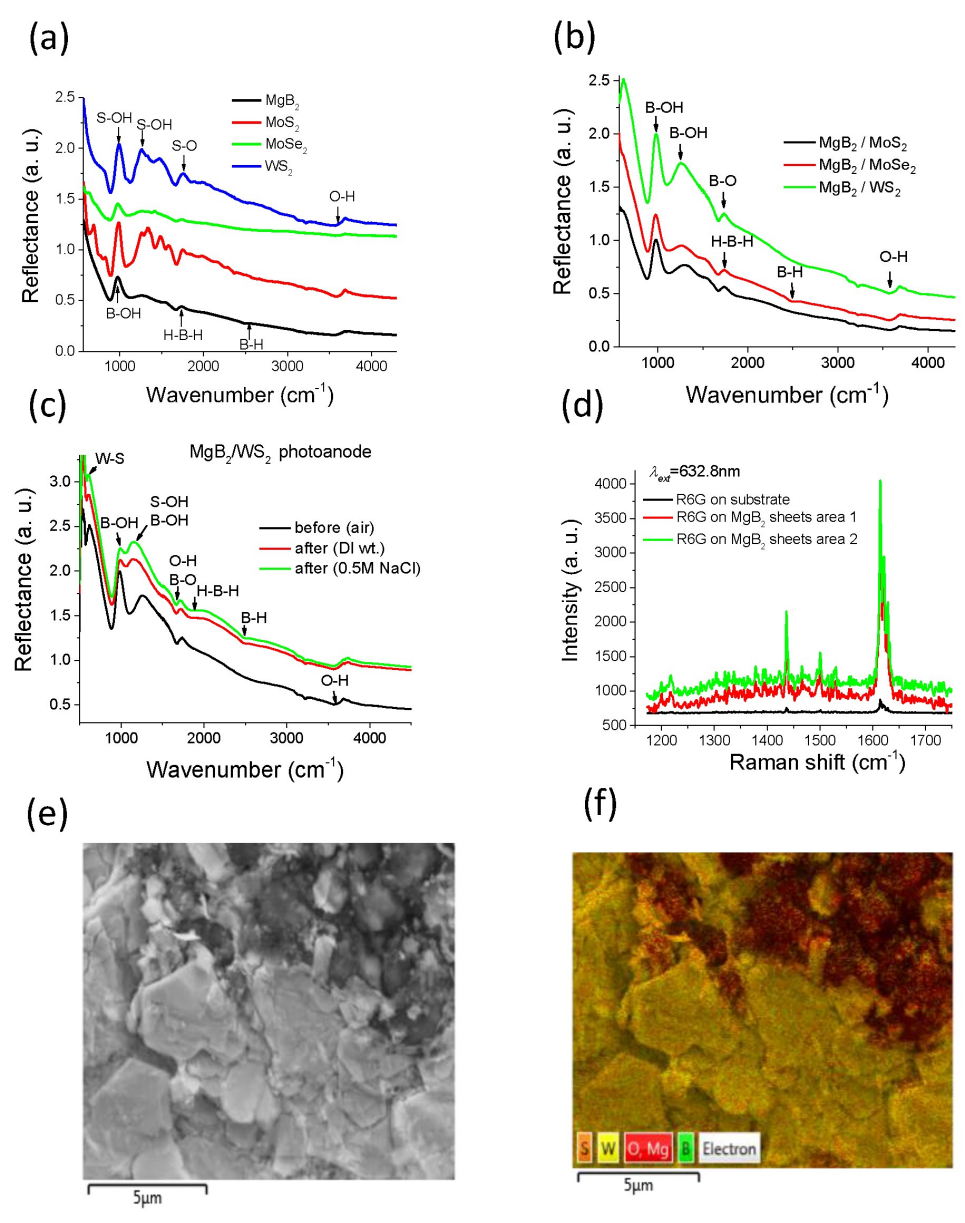
FTIR spectra measured on the studied photoanodes. (a) FTIR spectra taken from pure plasmonic MgB_2_ and TMDCs: MoS_2_, MoSe_2_ and WS_2_ layered nanostructures. (b) Comparison of FTIR spectra measured on MgB_2_‐TMDCs photoanodes. (c) FTIR measurements on plasmonic MgB_2_‐WS_2_ photo‐anode surface before and after water/seawater splitting experiments performed under *V_bias_* =0.3 V. (d) Surface enhanced Raman signals from R6G dye molecules placed on top of MgB_2_ nanosheet assemblies due to LSPRs of metallic MgB_2_ nanostructures that provide enhanced electromagnetic near‐fields (excitation with *λ*=632.8 nm). Energy dispersive X‐ray (EDX) analysis of a plasmonic MgB_2_‐WS_2_ photo‐anode surface. (e) SEM image of the analysed area; (f) EDX image of element distribution in the analysed area.

To study the properties of fabricated MgB_2_‐TMDCs photo‐anodes further we make use of the IR spectra of the samples measured with the help of Fourier Transform Infrared (FTIR) spectrometry. FTIR spectra provide information on bonding of H and OH water ions to the surface by registering vibration frequencies of various bonds. Appearance of such bonds between metal/semiconductor material and water molecules could provide an explanation for the efficiency of MgB_2_‐TMDCs photo‐anodes in photocatalytic seawater splitting. FTIR spectroscopy was performed using a Bruker Vertex 80 system with a Hyperion 3000 microscope. The measurement of the mid‐IR reflection spectra was done at the normal incidence at room temperature in the frequency range from 550 to 5000 cm^−1^ by performing 512 scans with a resolution of 4 cm^−1^ using cryogenic MCT detector cooled with liquid nitrogen. A gold thick mirror was used as a reference. The standard 15x vis‐IR (*N_A_* =0.4) Schwarzschild objective was used for focusing incident light on the surface of sample. As displayed Figure 4a, few reflection peaks appear for pure MgB_2_, MoS_2_ (MoSe_2_, WS_2_) samples.

The broadband absorption peak observed in the range 3400–3600 cm^−1^ corresponds to the water bending and indicates possible hydroxyl functionalisation. The peak at ∼981 cm^−1^ for MgB_2_ nanostructures corresponds to B−OH in‐plane bending and to a vibration mode at ∼1671 cm^−1^ associated with B−O stretch. The band at ∼1118 cm^−1^ can also be attributed to the presence of B−OH functional groups as the B−OH in‐plane bending is usually observed to be around 1000–1300 cm^−1^.[^33^, ^44^] The infrared spectra of the MoS_2_ (MoSe_2_) sheets exhibit strong bands around of 690 (663) cm^−1^ arising from Mo−S (Se) vibrations Figure 4a. These modes are non‐degenerate active modes of the first order occurring due to in‐plane vibrations normal to *c*‐axis.^[45]^ The observed peaks at ∼1200–1500 cm^−1^ are most probably connected to the S(Se)−OH bond vibration modes that confirm the appearance stretching modes of OH groups at the surface of MoS_2_ (MoSe_2_, WS_2_) sheets originated from air.^[46]^ The appearance peaks and their energy positions for MgB_2_‐TMDCs nanostructures are almost identical for three different samples, Figure 4b. The peak at ∼981–987 cm^−1^ for the studied photo‐anodes may correspond to a B−OH or S(Se)−OH in‐plane bending and vibration mode at ∼1671 cm^−1^ associated with B(S,Se)‐O stretch. The large IR absorption intensity ratio between the 981 and 1671 cm^−1^ peaks in the MgB_2_‐TMDCs nanostructures reflect the difference in the composition of samples and activity of corresponding vibrational modes. Small features also appear at ∼2500 cm^−1^ corresponding to absorption by the non‐bridging B−H groups (stretching vibration modes) at the sheet edges. The band at ∼1255–1300 cm^−1^ can also be attributed to the presence of B−OH functional groups in boron hydride B−H−S(Se) clusters. In MgB_2_‐TMDCs nanostructures activity boron to bond H and OH groups exceeds the ones of others elements such as S and Se. These results imply that proton release (H+) from the HB‐like sheets can occur to provide water splitting reaction. We found the B−H, B−H−B, and O−H vibrational signatures in the spectra of the investigated nanostructures, indicating that H of H_2_O can be exchanged with hydrogen of HB nano‐sheets.

Photochemical stability of MgB_2_‐WS_2_ hybrid nanostructures was examined by FTIR spectroscopy. Figure 4c demonstrates that solar irradiation caused no obvious change in the IR spectra of the MgB_2_‐WS_2_ nanostructures taken before and after water/seawater splitting. The fresh MgB_2_ sample displays bands that correspond to OH stretching at 3400–3700 cm^−1^ and water bending at ∼1670 cm^−1^ indicative of adsorbed moisture. FTIR spectra of MgB_2_‐WS_2_ sample have similar features after performing photocurrent measurements under bias of *V_bias_* =0.3 V in water/seawater environments. The surface of a MgB_2_‐WS_2_ photoanode exhibited an absorption band at ∼2485 cm^−1^, likely due to the B−H stretching and a band at ∼1585 cm^−1^ which can be ascribed to hydrogen motions in the B−H−B bridge. Strong absorption in the range of ∼980–1250 cm^−1^ is characteristic of B−O (S−O) stretch and hence the band at 1163 cm^−1^ can be assigned to the presence of oxy‐functional groups on the boron (sulphur) lattice. FTIR data suggest that the majority of water molecules are H‐ or HO‐bounded to the surface. The FTIR bands at 630 cm^−1^ shown in Figure 4c is attributed to the W−S stretching vibrations.^[33]^ Note that FTIR study confirms the absence of chlorine production on the surface of MgB_2_‐WS_2_ films. According to Huber,^[47]^ vibration modes associated with Mg(W)−Cl should be expected at <700 cm^−1^. They were not observed in the reflection spectra (Figure 4c). It is possible that a small amount of free chlorine (dissolved Cl_2_ or HClO) could be present but not recorded by FTIR. However, this amount should be negligible compared to the formed amounts of H_2_ and O_2_.

Scanning electron microscopy (SEM) images with energy dispersive X‐ray (EDX) analysis (Figure 4e and f) reveal a random distribution of different nanostructures with irregular shapes without any indication of specific morphology. EDX analysis of MgB_2_‐WS_2_ photo‐anode surface before and after photocurrent measurements under bias of *V_bias_* =0.3 V in seawater demonstrates the presence of Mg, B (contributions from the main catalyst) and W, S (contributions from the TMDC co‐catalyst) components in the both samples. EDX spectra also display small amounts of C, N and O embedded into the MgB_2_ or WS_2_ lattice and tiny amount of Na for photo‐anodes after seawater splitting. It is worth noting that the amount of detected Cl was very small (below the detection limit) indicating the absence of Cl corrosion. An increased amount of the O_2_ observed on the surface of MgB_2_‐WS_2_ after water splitting is probably produced by O_2_ bubbles during seawater splitting. Thus, the FTIR study of different vibration modes together with the EDX chemical surface analysis of photo‐ anodes after seawater splitting confirm the absence of chlorine production on the surface. This result shows that MgB_2_‐WS_2_ based nanostructures could be beneficial for seawater photocatalysis due to their strong resistance against corrosion of surface caused by Cl ions. We argue that the corrosion protection is based on the resistance of strong boron‐boron and boron‐metal bonds resulted from the large number of electrons surrounding the boron atoms.

## Discussion

A clear confirmation of LSPR excitations and hence plasmonic mechanism in photocatalytic seawater splitting based on the MgB_2_ nanostructures was obtained in studies of Raman scattering shown in Figure 4d. In these experiments, the fabricated MgB_2_ nanostructures were spin‐coated with a 10^−6^ M of R6G solvent in PMMA 950, 3 % anisole and Raman spectra were measured from the final system. The Raman measurements were performed using a Witec confocal scanning Raman microscope under a 632.8 nm laser light and a 1800 lines/mm grating with a x100 objective. The signal was collected from an area of 100×100 μm^2^. The laser incident power did not exceed ∼0.2–0.5 mW (to avoid damage or oxidation of MgB_2_ nano‐sheets and R6G molecules). Three characteristic peaks at 1437, 1502, and 1613 cm^−1^ are compared in Figure 4d for the cases of pure 10^−6^ M R6G on the Cu substrate, and a combination of MgB_2_ nanostructures and 10^−6^ M R6G dye layer. We found that the resonant Raman signal of R6G dye is enhanced by ∼20 times in magnitude by near‐fields of plasmonic MgB_2_ nanostructures, confirming the presence of LSPRs.

We found that the optical absorption of MgB_2_/MoS_2_, MgB_2_/MoSe_2_, and MgB_2_/WS_2_ photo‐anodes is enhanced in the visible‐light range (350<*λ*<650 nm) due to the composite structure of the anodes. The imaginary part of the refractive index, *k*, shows features associated with the bulk plasmonic peaks in the blue range (~400 nm) and a broad absorption region stretched from 500 to 1500 nm. The resonances in optical spectra shown in Figure 3 are due to specific morphology, i. e. shape, size, and configuration of MgB_2_ and TMDCs nanostructures where TMDCs absorb mainly ultraviolet‐visible light while MgB_2_ absorption extends into the red‐near IR range. As we explained above, a combination of metallic MgB_2_ and semiconductors MoS_2_ (MoSe_2_, or WS_2_) can provide the blackbody‐like optical properties for hybrid nanostructures submerged into seawater. Previously, an interesting observation of a broadband perfect absorption was observed in TMDCs of thicknesses ∼15 nm with Ag (Au) back reflectors.^[16]^ Similar features of MgB_2_‐TMDCs photoanodes could be very advantageous for off‐normal sun light harvesting. The photoanodes fabricated from MgB_2_ and MgB_2_‐TMDCs powders demonstrate visible and near‐IR plasmonic resonances, see Figures 1a, f–h. The multiple LSPRs of the fabricated photo‐anodes improve the absorption of light, enhance the excitation rate of the TMDCs excitons through local field enhancement, and provide generation of hot electrons in the metal‐semiconductor nanostructures[^48^, ^49^] improving the co‐catalytic activity of TMDCs. Therefore, the studied hybrid photo‐anodes effectively realise plasmon‐exciton co‐driven surface catalytic reactions for seawater splitting. We suggest the following reasons for effective seawater splitting with the studied MgB_2_‐TMDCs photo‐anodes: (i) absorption of light is enhanced by LSPRs of the hybrid photo‐anodes leading to increase of water splitting in the main MgB_2_ catalyst, (ii) these LSPRs induce intense electric fields and produce hot electrons in the corresponding TMDC semiconductor improving its co‐catalytic activity, (iii) MgB_2_ has negatively charged B layers and positively charged Mg layers which provide static electric field separating water ions produced during the water splitting.

**Figure 5:**
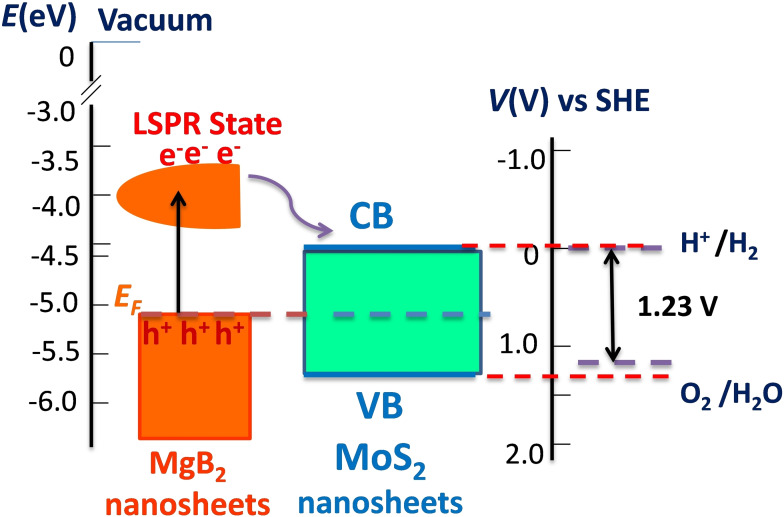
Schematic of the energy band diagram for plasmon‐enhanced seawater splitting based on MgB_2_‐TMDCs nanostructured photoanodes. Left: the Fermi level (EF) and hot electrons produced by LSPRs states for plasmonic MgB_2_ nanosheets. Middle: overview of the energy level positions of semiconductor VB and CB in contact with formation of a Schottky barrier between plasmon metal and 2D‐like TMDCs. Right: the electron potential (in eV) with respect to the redox potential of H+/H_2_ (0 V vs SHE) the redox potential of O_2_/H_2_O (1.23 V vs SHE).

A schematic model (Figure 5) shows plasmon‐induced photocatalytic reactions under LSPR excitations in hybrid MgB_2_‐TMDCs photo‐anodes (for concreteness, we consider MgB_2_/MoS_2_). Previously, it was found that MgB_2_ crystals with B‐terminated surface has a work function of *Φ_B_* ≈5.95 eV (∼1.45 eV vs standard hydrogen electrode (SHE) with *Φ_H_* ≈4.5 eV), MgB_2_ with the Mg‐terminated surface has *Φ_Mg_* ≈4.25 eV (−0.25 eV vs e$V_{H^{+}/H_{2}}$ =−4.44 eV or 0 V vs SHE) while the Fermi level is located at ≈5.1 eV.^[50]^ At the same time, the MoS_2_ flakes with thickness 10–30 nm has a band gap of 1.3 eV suitable for photon absorption from UV to NIR ranges.^[51]^ The electron affinity of MoS_2_ is around χ_MoS2_∼4.3 eV, while the work function of MoS_2_ is ∼4.7 eV. A Schottky barrier (SB) height between MgB_2_ and TMDCs can be calculated using the Mott–Schottky equation, which states that the barrier height is the difference between the work function of the metal and the electron affinity of the semiconductor: *φ*_B_=*φ*_MgB2_‐*χ*_MoS2_. Here *φ*_B_ is the Schottky barrier height, *φ*_MgB2_ is the work function of the MgB_2_ metal and *χ*_MoS2_ is the electron affinity of the MoS_2_ semiconductor. In Figure 5 the Fermi level gets pinned below the conduction band of MoS_2_ due to strong metal‐MoS_2_ interaction at the interface. Note that recent studies indicate that *φ*_B_ is not only dependent on the work function of the metal and semiconductor, but also dependent on the metal‐semiconductor interaction, which causes charge redistribution at the interface. Metal‐semiconductor interaction at the interface creates an interfacial dipole and results in pinning of the Fermi level just below the conduction band of the semiconductor.^[52]^ A low SB height promotes an increase of the transfer of hot electrons from the metal to TMDCs.[^12^, ^53^] The plasmon resonance frequencies of nanostructured photo‐anodes lie above 600 nm, see the absorption coefficient *k* shown in Figure 3. Thus, we can expect a maximum energy of ∼2 eV for hot‐electrons generated from the LSPRs. Such energetic electrons from plasmonic MgB_2_ nanostructures can be injected into the conduction band of TMDCs nanosheets (Figure 5). A hot electron mechanism of photocatalysis, see review,^[10]^ applied to our hybrid structures assumes the following: (i) Plasmons in MgB_2_ nanostructures generate hot carriers for photocatalysis. (ii) The energy of hot electrons could be transferred from MgB_2_ nanostructures to TMDC nanosheets to promote electron–hole excitation in WS_2_ (MoS_2_, MoSe_2_) and the efficiency reaches the maximum when the LSPR wavelengths of MgB_2_ nanostructures matches the WS_2_ (MoS_2_, MoSe_2_) light absorption bandgap. We do not know at present whether the hot electron mechanism could be applied to our studied hybrid photo‐anodes or some other mechanism is responsible for obserbed seawater splitting.

Ellipsometric measurements for thin layer MgB_2_‐TMDCs hybrid structures have shown that the enhancement of localised electromagnetic field due to the LSPRs of MgB_2_ can improve the light absorption of such systems, especially in water environment. The LSPRs of MgB_2_ component improve the water splitting by MgB_2_ and also enhance the excitation rate of MoS_2_ excitons through local field enhancement.^[51]^ The plasmonic MgB_2_ surface acts a charge‐trapping hub significantly inhibiting the recombination of electron‐hole pairs, which is crucial for realising photo‐catalysis devices with more efficient solar‐energy conversion.[^23^, ^24^] Hence, the MgB_2_‐TMDCs hybrid photo‐anodes effectively improve the light‐harvesting efficiency of pristine 2D‐like TMDCs owing to strong plasmon–exciton coupling, the hot electrons transfer from the plasmonic metal to TMDCs, and separation of ions produced by water splitting due to the MgB_2_ surface. The optical absorption of MoS_2_, MoSe_2_, and WS_2_ materials is large in the visible‐light range (300–700 nm) with attenuation coefficients reaching 10^5^–10^6^ cm^−1^ due to small bandgaps (*E_g_* <1.5 eV). However, the overall efficiency of pure TMDCs (without plasmonic contributions of MgB_2_) is not large as only the edges of flakes work as effective co‐catalysts.[^54^, ^55^] The best performance of TMDC thin film photoanodes for water splitting demonstrates an overall efficiency of approximately <1 %, for review see.^[56]^ Finally, we address the point of thermal effects that could be possible in the studied photoanodes due to strong light absorption. LSPRs may lead to local heating under light illumination. There are several observations demonstrating that heating of water on hot spots is not responsible for the studied water splitting. First, we observe formation of bubbles on both electrodes: the bubbles on a smooth Pt wire cannot be explained by plasmonic LSPR effect. Second, the overall amount of bubbles on both electrodes strongly increased when an increase of the bias voltage (which would not be the case if the bubbles were produced by water heating induced by the solar simulator). Third, we performed experiments in a heated electrolyte with MgB_2_‐WS_2_ photo‐anode and Pt cathode (reaching temperatures up to 80 °C). These experiments showed a decrease of efficiency of photocatalytic water splitting.

## Conclusions

In conclusion, we have shown that metal‐semiconductor metamaterials can be an effective photocatalysts for seawater splitting. The novelty of these exceptional metamaterials comes from the fact that Earth‐abundant metal (MgB_2_) is used to initiate the plasmonic water splitting reaction while TMDCs are used to improve light absorption of photoanodes and to serve as co‐catalysts. The overall conversion of light to hydrogen by our catalysts was ~3 %. We also suggested a new method of fabricating large area plasmonic metal‐2D semiconductor photo‐catalysts. The method is based on a mechanical rolling mill procedure and can be easily implemented by the industry. The fabricated photoanodes consist of randomly distributed metallic MgB_2_ and TMDCs nanostructures and provide better efficiency than photo‐anodes produced from individual components. The complex refractive index of the MgB_2_‐TMDCs metamaterials has been measured and confirmed the presence of multi‐wavelength LSPRs and exciton features in the assembled systems. Fabricated MgB_2_‐TMDCs hybrid photoanodes demonstrate improved photon absorption in broad spectral range due to strong plasmonic resonances of MgB_2_ nanostructures in the visible –near IR light. The photocatalytic efficiency of the photo‐anodes in seawater splitting was improved by fabricating composite photo‐anodes with refractive indices that lead to plasmonic blackbody behaviour. We found that the photocurrents for MgB_2_/WS_2_ photo‐anodes are ∼9 times larger than those for bare WS_2_ and ~4.5 times larger than those for pure MgB_2_ photo‐anodes. We showed that non‐noble metal plasmonic MgB_2_ combined with TMDCs could allow one to overcome limiting factors of photocatalytic efficiency existing for broad bandgap semiconductors by using the whole range of the solar spectrum that could drive the photocatalytic water splitting reactions. The IR measurements provided additional information on the charge transfer suggesting that the B−O−H bonds stretching could play an important role in plasmon enhanced seawater splitting.

## Author Contributions

VGK and ANG initiated the project. VGK and HZ fabricated devices, performed photocatalytic tests and ellipsometric measurements. VGK carried out the FTIR measurements. ANG analysed the data. All authors discussed the results and contributed to writing the paper.

## Conflict of Interests

The authors declare that there are no conflict interests.

1

## Data Availability

The datasets generated during and/or analyzed during the current study are available from the corresponding author on reasonable request.
